# Supplementary material for: Acceptability and feasibility of the NPS MedicineWise mobile phone application in supporting medication adherence in patients with chronic heart failure: Protocol for a pilot study
Source: PLoS One. 2022 Feb 4;17(2):e0263284. doi: 10.1371/journal.pone.0263284 (PMC8815969; doi:10.1371/journal.pone.0263284)
Supplement: S1 File — (DOCX) [file pone.0263284.s001.docx]

**Title: Medicine adherence in chronic heart failure**

**Investigators:**

**University of South Australia: Dr Vijay Suppiah**

**Dr Elizabeth Hotham**

**NPS MedicineWise: Ms Nerida Packham**

**Site PIs: Dr Alicia Chan (The Royal Adelaide Hospital and The Queen Elizabeth Hospital)**

**A/Prof Margaret Arstall (Lyell McEwin Hospital)**

**Dr Christine Burdeniuk (Flinders Medical Centre)**

**Dr Genevieve Gabb (Noarlunga GP Plus)**

**Ms Teena Wilson (ICCNet)**

**Associate Investigators: Mr Jeffrey Briggs**

**Mr Tim Pearson**

**Mrs Terina Selkow**

**Mrs Adaire Prosser**

**Miss Jessica Chapman-Goetz (Study RA; Masters student)**

**Introduction**:

Heart failure (HF) is a complex clinical syndrome characterized by the reduced ability of the heart to pump and/or fill with blood, causing a significant burden for patients and healthcare systems in developed countries. HF is increasing in prevalence affecting at least 26 million people worldwide (Savarese & Lund, 2017). HF accounts for approximately 1-3% of overall healthcare spending, mainly due to repeated hospital admissions and prolonged inpatient length of stay (Sahle et al., 2016). However, the return of investment in healthcare spending in HF is very poor as studies suggest that approximately 50–75 % of patients with HF die within five years of diagnosis and this expenditure has been estimated to increase dramatically with an ageing population (Sahle et al., 2016).

Despite the significant advances in therapies and prevention, non-adherence with medication in HF patients is associated with excess mortality and morbidity. Medication adherence is defined as the extent to which a patient takes medications as prescribed by their healthcare providers (Chowdhury et al., 2013). While the World Health Organization describes poor adherence as ‘a worldwide problem of striking magnitude,’ it is a problem that should be viewed as ‘diagnosable and treatable’ (Marcum et al., 2013; WHO, 2003).

A systematic review estimated adherence to medications for HF to be 48.4% (95% CI 9.0% to 89.2%) (Bowry et al., 2011). Suboptimal adherence is recognized as a global challenge to health systems, due to its strong association with an increased risk for poor health, adverse outcomes and mortality (Forsytha et al., 2019; Hines et al., 2018). For example, Chowdhury et al. estimate that of all adverse medication reaction-related hospital admissions in the USA, 33–69% are due to poor medication adherence with an estimated cost of $100 billion (Chowdhury et al., 2013).

While Bowry et al. (2011) report that adherence does not significantly change according to gender, age or the complexity of medication regimens, factors which significantly influence adherence levels are low social status, low health literacy, existence of co-morbid conditions, and polypharmacy (Bowry et al., 2011). Other common predictors of poor adherence include negative perceptions about medication, side effects and high medication costs (Bowry et al., 2011). Furthermore, self-efficacy for appropriately taking HF medications may be predictive for physical health status where individuals with higher self-efficacy report better physical health status, including symptom control, and quality of life. Clinically, higher self-efficacy has been found to improve cardiac function, which may explain improvements in physical health status as symptom burden is less. Consequently, supporting people’s self-efficacy and confidence in their selfcare activities will impact their medication adherence, condition control and health status (Como 2018).

The use of the internet and smartphones to deliver health care is growing rapidly (Becker et al., 2013). Mobile health (mHealth) interventions, such as smartphone applications (apps), have been advocated as promising strategies to assist in the self-management of hypertension and other chronic conditions. The number of smartphones worldwide is predicted to reach 5.8 billion by 2020 and there are 6 million multimedia applications (apps), of which 318,000 are mHealth apps, available for download in the app stores (Byambarsuren et al., 2018). With the global prevalence of mobile technology, accessing health-related applications via mobile phone seems a logical step in the patients’ management of their own medical conditions. While there is still a threefold ‘‘digital divide’’ between age groups, sexes and according to education levels, it seems likely that mHealth apps will increasingly play a role for elderly and chronically ill users (Becker et al., 2013). However, guidance and coaching by the healthcare provider will be necessary and not overloading an application with functionality is essential when developing for elderly users with chronic conditions (Becker et al., 2013).

Electronic devices are increasingly used by both health care providers and patients as communication tools. Providers using mobile communication technology to connect with their patients achieve an improved overall patient-provider communication, strengthened patient autonomy, and empowered patients to tackle daily health issues (Becker et al., 2013). Studies from various clinical contexts, in which text-messaging services were introduced, reported improved medication adherence (Morawski et al., 2018; Becker et al., 2013). mHealth tools have the potential to address nonadherence by providing reminders for medication taking and refilling, tracking biometric results, offering education and facilitating social interactions that provide support and motivation. By assisting the patient to actively measure, monitor and manage their health condition, mHealth apps promise to improve health outcomes.

While technologies, such as smartphone apps, are promising tools to help reduce the global problem of non-adherence to long-term medications further evidence is needed to help guide health professionals and health services. The present study aimed to determine whether a personalized progressive reminder system using the NPS MedicineWise app will improve medication adherence in HF.

**Hypothesis**:

The primary hypothesis is that reminders via a smartphone application with a personalized and progressive monitoring system plus usual care (as received by participants in the intervention arm) compared with usual care alone (as received by participants in the control arm) will be associated with higher medication adherence, at 9 months’ follow up.

The secondary aims of this trial includes:

1. to determine whether a smartphone application reminder with interactive and personalised monitoring is cost effective, as determined by lower health services utilization, at 9 months’ follow up;

2. to determine the impact of the smartphone application on signs and symptoms of heart failure and quality of life at 6 and 9 months;

3. to determine whether the intervention is associated with higher medication and heart failure knowledge;

4. to determine the feasibility, acceptability and engagement with the application as determined by the

**Methods and Analysis**

This protocol is reported in accord with the Standard Protocol Items: Recommendations for Interventional Trials (SPIRIT) 2013 guidelines for protocols of clinical trials (Chan et al., 2013).

**Design**

The study is a prospective, single-blind observational RCT with individual participant allocated to the intervention or control group (Figure 1). Participants randomized in the control arm will be blinded to the intervention. Researchers undertaking the data collection will not be blinded due to the personliased and progressive monitoring nature of the intervention arm.

**Setting**

The study will be conducted in six investigator centres in South Australia: five South Australian metropolitan hospitals (Royal Adelaide Hospital, Queen Elizabeth Hospital, Lyell McEwin Hospital, Flinders Medical Centre and Noarlunga Hospital) and rural patients will be recruited from integrated Cardiovascular Clinical Network (ICCNet). A total of 220 patients will be recruited and randomized to either the intervention arm or control arm of the study. Eligible participants will be briefed about the study by the treating physician or nurse and they would then be referred to a member of the research team either in person or by skype call when participants come into the hospital for their regular outpatient clinic visits, when the community cardiac nursing team visit patients at home or, in the case of rural patients, during their scheduled skype ‘visit’ with cardiac nurses. Potential participants will be provided with a summary study information and will be contacted by the research team for an initial face-to-face interview either at any of the participating hospitals or via skype for rural patients. At this initial visit, after screening for eligibility, written informed consent will be obtained from those who agree to participate in the study and a baseline assessment will be completed by a member of the research team. A record of non-participation will be kept for those who are ineligible or those who decline to participate in the study. Follow ups at months 3, 6 and 9 will be conducted over phone or skype by a member of the research team.

**Study population**

Entry criteria are as follows: (i) age 18 years or older and able to give written informed consent; (ii) confirmed diagnosis of systolic heart failure on echocardiography (iii) NYHA functional class I-III for at least 3 months; (iv) Left ventricular ejection fraction (LVEF) < 50 % at or around the time of diagnosis of heart failure in keeping with the 2018 Australian Heart Failure guidelines (which will also include patients with an improved, or recovered ejection fraction of >50% following treatment)(Atherton et al. 2018) (v) stable or stabilized condition (vi) Participant or carer with access to a smartphone or similar smart device and have adequate access for receiving and responding to emails and skype calls.

Exclusion criteria includes: (i) palliative heart failure (ii) NYHA functional class IV (iii) malignancy or diastolic heart failure (iv) unable to understand the study information or unable to complete outcome questionnaires (ie. if an interpreter is required) (v) life expectancy less than 6 months or patients engaged with the palliative care network (vi) use of other medication reminder app or other electronic reminder systems for daily administration of medications; (vii) participant or carer who do not have access to a smartphone or similar device or do not have adequate access for receiving and responding to emails and skype calls.

Participants are free to withdraw from the study at any point in time and this will not impact their on-going care. This will be emphasized during the consent process. If a participant chooses to withdraw, the reason for withdrawal will be requested and recorded. All data collected to the point of withdrawal will not be included in the final analysis.

**Recruitment of participants**

i. Site PIs (cardiologists) and cardiology nurses and cardiology pharmacists will identify potential study participants. Cardiologists, cardiology nurses and cardiology pharmacists have been briefed about this study and they are aware of the inclusion/exclusion criteria. They have been requested to refer patients whom they are seeing in their clinics and pharmacies to the study team. As a reminder of the study, all clinicians will have a clinician flyer which will be put up at a spot where they can see it. At this stage of the recruitment process, the study team will not have any access to potential study participants.

ii. During the referral process, site PIs (cardiologists) and cardiology nurses and cardiology pharmacists doing the referral will brief their patients about this study and provide them with a study flyer. Details of potential participants will be provided to the research team by the referring clinicians via email. The email by the clinician will also give permission to the research team to have access to the potential participants medical records should they give informed consent to participate in the study.

iii. A member of the research team will contact potential participants referred to them by the clinicians one week later for an initial interview. At this initial interview, the member of the research team will screen the potential participant for eligibility and then obtain informed consent from those who agree to participate in the study. This contact will be in the form of phone call, via skype (mainly for the rural patients) or face to face if the potential study participant is coming into the clinic for one of their routine follow ups.

iv. Only after getting informed consent from study participants will the member of the research team conduct a baseline assessment which will also consists of getting clinical data from the patient’s medical records.

**Randomisation and blinding**

Participants will be randomly allocated in a 1:1 ratio to either intervention or control group arms. Randomising of study participants will be done according to the random number generator from <https://www.random.org/integers/?num=220&min=1&max=220&col=5&base=10&format=html&rnd=new>. Participants allocated even numbers will be cases and participants allocated odd numbers will be controls. Participants randomized in the control arm will be blinded to the intervention. Due to the nature of the intervention, it will not be possible to blind the investigators. However, to minimize bias, all participants will be allocated a study ID and all data will be collated using the study ID. Analysis of the data will be conducted by blinded investigators.

**Intervention**

The intervention was developed based on the principle of cued actions of the Health Belief Model (Strecher and Rosenstock. 1997). This model states that external cues, such as reminders and prompts, may increase adherence behaviours.

Usual care arm

Participants randomised to the usual care arm will receive standard care for their HF as determined by their treating physicians, including prescriptions to treat CHF and advice, as well as lifestyle advice. The participants in this group will have no access to a smartphone medication reminder application.

Intervention arm

Participants allocated to the intervention arm will have access to the NPS MedicineWise application (MedicineWise app) in addition to standard care (as described above). Once consented, each participant in the intervention arm will be given training by a member of the research team. This training will include how to download the MedicineWise app from the participant’s smartphone application store, navigate the features within the MedicineWise app, how to enter all of their current medications (both CHF related and non-related) into the app (including medication name, strength, dosage form, dose and frequency of administration), how to edit or delete a medication if there are instructed changes from their treating physician, how to set up in-app reminders, how to acknowledge and register a taken dose and troubleshooting and technical support information. The researcher will also provide practical tips to ensure participants can get the best experience from their interactions with the app. instruct participants in the intervention arm how to edit or delete a medication if the medication, dose or timing is either changed or suspended by their treating physician. At the end of the training, participants will be asked to demonstrate to the researcher team member that they can perform these functions on the app comfortably and that they know where and how to seek help should any problems occur.

Tier 1 intervention: in-app medicine dose reminders from the MedicineWise app

Once downloaded and all medication related information has been uploaded onto the application, participants will receive in-app reminders (in the form of notifications and audible alarms, if they choose) when each medicine dose is due to remind them to take their dose. Participants have the option to dismiss or “snooze” the reminder. Dismissing a reminder means that the participant will not receive that specific dose reminder again. “Snoozing” a reminder means the reminder would then be repeated at default 30-minute intervals (the default will be adjusted according to the participant’s preference to truly reflect real-life use of this snooze function). Participants can acknowledge that they have taken their medicine doses in the app by tapping on a “taken” button that is associated with the dose. Information of either the dose being recorded as taken or missed (i.e. not marked as “taken”) will be registered by the MedicineWise app and will be extracted daily at NPS MedicineWise headquarters. A NPS MedicineWise research team member will determine from the extracted data the doses that were missed during the 24 hours prior, according to the criteria set for critical or non-critical medications (see Tier 2 intervention).

Tier 2 intervention: personalised ‘push’ notification messages from the MedicineWise app

Medications have been categorised as critical or non-critical to the management of heart failure (Table 1). If a participant has either missed a dose of a critical medication for 24 hours or a non-critical medication for 3 days, they will receive a personalised ‘push’ notification from the MedicineWise app containing one or more of the following messages: reminding them to take their doses; reminding them to interact with their app; emphasizing the importance of adhering with their medication regimen; how to seek help if they are having difficulty with their medication and/or operating the MedicineWise app.

In the same way as for Tier 1 escalation, a NPS MedicineWise research team member will examine the extracted dosage taken data during the 24 hours prior and escalate participants to the Tier 3 program as required.

Tier 3 intervention: phone call intervention delivered by Medicines Line pharmacists (NPS MedicineWise)

Participants who do not respond to MedicineWise app notifications (Tier 1 intervention) and ‘push’ notification messages (Tier 2 intervention) will be contacted by a NPS MedicineWise’s Medicines Line pharmacist by telephone to discuss their medicines and medication adherence. A NPS MedicineWise research team member will provide the Medicines Line pharmacist with the participant’s details and records in a secure file in a shared drive with password access. Only the NPS MedicineWise research team members and Medicines Line team will have access to this file.

The Medicines Line pharmacist will contact the participant by telephone, as soon as possible (ideally within one working day of the participants’ escalation into the Tier 3 intervention) and record details of the call in MiDatabank® software. Keyword ‘heart failure trial’ can be used to identify calls when retrieving data. Data can be extracted from the Midatabank software to assist with analysis. Five attempts at different times of the day and different days of the week will occur. Details of attempt time and date will be recorded.

Once telephone contact is made, the following details will be recorded in MiDatabank:

- Particpant name, date of birth, postcode, gender, ethnicity, phone number, ‘heart failure trial’ keyword.
- Consent documented to advise UniSA Research Assistant that call has occurred.
- Current medications: medicine name, brand, strength, dosage.
- Details of conversation with patient.
- Details on adherence, including possible reasons for non-adherence eg.side effects, concerns with drug interaction or other reasons – keywords to be documented in MiDatabank
- Success or difficulties with using the MedicineWise application.
- Details of medicine information resources accessed.
- Evidence-based medicine information can be provided using standard resources, including but not limited to:
- Therapeutic Goods Administration Product Information and Consumer Medicine Information
- MIMs
- Australian Medicines Handbook
- Stockleys Drug Interactions
- Details of discussion with patient and information provided, including discussion on importance of heart failure medicines and how to improve adherence. Risk factors for reducing complications of heart failure can also be discussed and documented.
- Patients can be referred to their doctor, specialist or community pharmacist, if clinically indicated.

A participant can be contacted up to a maximum of 3 times by a Medicines Line pharmacist during the trial to discuss medication adherence. Each call will be documented in MiDatabank. Details (including the patient’s name, date of birth, MiDatabank keywords, call category) for each call will be forwarded via secure digital pigeon, with password access, to the research team member at the University of South Australia to inform of the intervention.

Tier 3A intervention: personalised ‘push’ notification messages from the MedicineWise app during weekends

The Medicines Line is an office hour service which will be operative Mondays to Fridays 9 am to 5 pm AEST (excluding NSW public holidays). This is a ‘real world’ intervention. Out of these hours, participants will receive a further personalised ‘push’ notification which will be followed up on Monday morning. If participants are concerned about their medications during the weekend, they will be advised to follow their standard medical care plan and contact their own doctor, pharmacist or emergency services if applicable.

Other features of the MedicineWise app

Other features within the MedicineWise app, includes the ability to have multiple profiles (carer mode) and the ability to record health conditions, allergies, test results and other health information. Recent enhancements have also enabled the app to deliver featured health- and medicine-related content to users based on their medicines and/or health conditions. Participants in the intervention arm will be able to use any of these features within the MedicineWise app; however such additional data collected by the app will not be used for the purposes of the current study.

**Outcome measures**

Outcome measures will be collected at 3, 6 and 9 months following the baseline data collection at recruitment.

Primary outcome

The primary outcome is the comparison of medication adherence between the two arms. Medication adherence will be measured by self-reported medication adherence tool Self-Efficacy for Appropriate Medication Use Scale (SEAMS) (Risser et al., 2007). This has been validated previously in other cardiac conditions such as coronary heart disease (Risser et al., 2007). This self-reported data will also be verified by the data collected from the intervention arm participants whose adherence rate has been captured by the data collected by the NPS MedicineWise application.

Secondary outcomes

i. composite outcome of death or hospital admission related to HF or not related to HF. All instances of hospitalization and death will be recorded and classification as to whether it was HF related or not will be done by experienced cardiologists

ii. health resource utilization (ie. Primary and secondary care contacts, social care contacts and relevant medication use)

iii. health related quality of life, assessed by EQ-5D-5L

iv. self-reported medication adherence

v. assessment of exercise tolerance (6 min walk test)

vi. psychological wellbeing (Depression anxiety and stress scales)

vii. self-care of HF index questionnaire

viii. medication knowledge

ix. acceptability, utility and engagement with the NPS MedicineWise application (Satisfaction survey)

**Data that is captured by the MedicineWise app**

The dataset for each patient in the intervention arm can be generated on a daily basis and exported in a spreadsheet format and can be used by the project team. The type of raw data that is captured is divided into four categories and described below. Further manipulations of this raw data may be necessary (e.g. to identify the participant and to calculate the number of doses missed) and will be carried out as part of the analysis in this study.

The 4 fields of data are:

1. **User ID** – the data extracted from the app will be a unique key that will then need to be de-identified to reveal the participant’s name, DOB, sex and email address. There will be a map provided at the beginning of the study once the participants have been recruited has downloaded and have registered with the MedicineWise app that will enable this process to occur, and a simple Excel matching function should make things easier
2. **Medicine details** – this will include all the medicines that the participant has on their medicines list in the MedicineWise app that are on the critical and non-critical list, and it would include the name of the medicine, strength and form.
3. **Dosage and frequency**– this will include how the patient has been instructed to take their medicines, as entered in the app. In addition to the dose and frequency, it will also include specific times that doses are due.
4. **Actual time when doses are recorded as taken**– when the doses are recorded as taken, timestamp of when that occurs is recorded by the MedicineWise app. The participant has the ability to change that time if they wish (e.g. if a medicine dose is due at 8am and took their dose at 8.30am but they only marked their dose as being taken at 6pm, the app will record the time taken as 6pm but the patient has the ability to change this back to 8.30am). The difference between the time taken and when the doses were supposed to be due will be calculated and used monitor medication adherence.

**Sample size**

Sample size calculations are focused on two different measures and were also done assuming equal numbers of participants in each group. The first measure takes into account an absolute increase in mean adherence of 15 % (Hulley et al., 2013). In this measure, at two tailed 0.05 significance level with a power of 80 % and standard deviation of 35 (Goldstein et al 2014), the number of participants required in each group is 86. In the second measure, the analysis is by comparing proportions of adherence (Hulley et al., 2013). Previous studies have estimated the adherence to cardiovascular drugs to be up to 60 % (Chowdhury et al., 2013; Bowry et al., 2010). In this measure, a total sample size of 182 participants is needed to provide 80 % power to detect an increase in the proportion of adherent participants from 60 % to 80 %, with a two tailed 0.05 significance level. Therefore, taking the larger of the two sample sizes (91 participants in each arm), and allowing for a 20 % loss to follow up, a total of 220 participants will be recruited in this study with each arm consisting of 110 participants.

**Trial data collection**

Trial data will be collected at 4 time points (at baseline, 3, 6 and 9 months). Data will be collected at face-to-face during home visits, phone calls or via skype interviews (mainly for rural participants). To encourage participant retention and completeness of data, participants will receive a honourarium of AUD$30 in total at completion of the study.

At the point of recruitment into the trial, baseline data will be collected after written informed has been obtained by a member of the research team. The following information will be collected:

Sociodemographic information: Date of birth, gender, ethnicity, height, weight, education status, marital status, employment status, smoking status and health literacy

Medical history: comorbidities, NYHA class, standardised mini-mental state exam score, depression score, all prescribed and non-prescribed medication history

Laboratory test results: full blood counts, full electrolytes (sodium, potassium, urea, creatinine +/- magnesium), Hb, iron studies, NT pro BNP, liver function tests.

Functional assessment: QoL questionnaire using the Short Form 36 Health Survey version 2 (SF-36v2) (Ware JE; RAND Corporation 2015), Depression Anxiety and Stress Scales (DASS), Self-Care of Heart Failure Index (SCHFI) (Riegel et al., 2009), EQ-5D-5L questionnaire (Herdman et al., 2011) and drug adherence questionnaire, healthcare utilization (linkage data from Australian Pharmaceutical benefits Scheme and Medicare Benefits Schedule).

At the 3, 6 and 9 month follow ups, a member of the research team will record details of any changes to the participants’ medication or implantable cardiac devices, details of any hospitalisations or healthcare resource utilization since the last follow up. At each follow up interview, participants in the control arm will be asked whether they have used any drug administration aids or medication reminder app or other forms of electronic reminder systems for daily administration of medications. At the end of the study, intervention arm participants will complete a satisfaction survey to assess the acceptability, utility and engagement with the NPS MedicineWise application. Also, data about prescription refill rates and healthcare utilization will be gathered for all participants through data linkage with the Australian Pharmaceutical benefits Scheme and Medicare Benefits Schedule.

Data will be collected at each follow up on forms and then recorded as de-identified data only identified by the participant’s study number and stored electronically. At each follow up time point, 10 % of the data will be randomly selected for verification. In the event that discrepancies are identified, these will be verified using the original paper data sheet.

Participants’ names, addresses and smart phone numbers will be collected for the purpose of intervention delivery and participant interviews. All data collected from participants will otherwise be stored electronically as de-identified data which will be stored separately from identifiable data. All paper based data forms will be stored in locked filing cabinets within offices with restricted access. Electronic records will be stored in a secure server maintained by the University of South Australia. Data in the database will be backed up regularly. Access to all data will be restricted to the members of the research team and data will not be shared with any third parties.

**Process evaluation**

**Economic evaluation**

A rigorous and systematic approach to the economic analysis will be applied applying international standard best practice methods for the design and conduct of economic evaluations in health care (Drummond et al., 2015; Brazier et al., 2016). The cost-effectiveness of the proposed intervention will be estimated using a cost-utility analysis (CUA) undertaken alongside the RCT with quality adjusted life years gained (QALYs) as measured by the EQ-5D-5L as the main measure of outcome. The purpose of the economic evaluation is to compare the differences in the streams of outcomes and resource use that will occur as a consequence of the introduction of the mobile phone app. This will be expressed in terms of the incremental cost per QALY gained in the Australian setting. A health system perspective will be taken for the economic evaluation including health related resource use (costs and cost-offsets) and health related quality of life outcomes. As the economic evaluation will be conducted alongside the clinical trial with a 9 month follow up period, discounting of costs and benefits beyond the base year of the economic evaluation will not be necessary. The setting of the economic evaluation will be the Australian health care setting.

The intervention will be tested in the RCT in the circumstances in which it will be expected to be applied should it be adopted for wide-spread routine application within the health system. The main clinical outcomes of the RCT encapsulate directly measured and reported participant relevant end points. The follow up period of 9 months is an appropriate time horizon within which all patient relevant end points will be adequately captured.

**Data analysis**

The primary analyses will be based on a between-group (intervention arm vs control arm), intention-to-treat approach at the different time points. Continuous variables will be analysed using independent t-tests and discreet variables will be analyses using chi-squared tests. Where appropriate, other statistical tests will be employed depending on the nature of the data. Identification of any confounding baseline variables will be corrected by logistic regression analysis. All between group outcome results that are significant will be reported with odds ratios and 95 % confidence intervals. Statistical significance is set at α 0.05.

Qualitative outcomes

Thematic analysis will be carried out based on patient responses to open-ended questions in the functional assessments. This will enable to generate emerging and overarching themes. The analysis will characterise intervention arm participants’ observed and self-reported responses to the intervention and link these responses to the acceptability, utility and their engagement with the smartphone app. This thematic analysis will be based on the intervention arm participants’ responses collected at months 3 and 9, in addition to a satisfaction survey undertaken upon completion of the trial.

Economic outcomes

Data on the frequency and duration of hospital in-patient admissions will be obtained from medical records, and PBS and MBS utilisation data from Medicare following informed patient (or proxy in the case of moderate to severe cognitive impairment) consent. Unit costs will be derived from relevant hospital finance departments, published data sets including Pharmaceutical Benefits Scheme and Medicare Benefits Schedule and Australian Refined Diagnosis Related Groups (AR-DRG) cost weights.

A participant level analysis will be undertaken to determine the incremental costs and outcomes associated with the intervention relative to controls. Incremental cost effectiveness ratios and their associated confidence intervals will be estimated and cost effectiveness acceptability curves for varying threshold values of cost effectiveness will also be presented. An assessment of the sensitivity of the results obtained to variation in measured resource use, effectiveness and/or unit costs will be undertaken using appropriate one-way, multi-way and probabilistic sensitivity analysis.

**Ethics and dissemination**

The study will be conducted in accordance to the ethical principles of the Declaration of Helsinki and will adhere to the National Health and Medical Research Council ethical guidelines for human research. The study has been funded by VentureWise Pty Ltd and the Department of Industry, Innovation and Science. Ethical approval has been obtained from the Central Adelaide Clinical Human Research Ethics Committee and University of South Australia Human Research Ethics Committee. Written informed consent will be obtained from all participants prior to study enrolment. Additional individual informed consent for data linkage through the Australian MBS and PBS will also be obtained. Australian New Zealand Clinical Trials Registry Clinical trial registration number: ACTRN12619000289112p (http://www.ANZCTR.org.au/ACTRN12619000289112p.aspx)

Findings will be published in peer-reviewed journals and presented at local, national and international meetings and conferences to publicise and explain the research to clinicians and end-users.

**Conclusion**

This randomized controlled trial aims to examine the addition of NPS MedicineWise application based progressive reminder system to standard care on adherence to daily medication for the treatment of HF. The study will provide valuable information for clinicians, policymakers, patients and their caregivers about the role of smartphone applications as a reminder system to increase medication adherence and has the potential to reduce unnecessary healthcare utilization due to lack of adherence in heart failure.

References:

Atherton JJ, Sindone A, De Pasquale CG, Driscoll A, MacDonald PS, Hopper I et al., National Heart Foundation of Australia and Cardiac Society of Australia and New Zealand: Australian clinical guidelines for the management of heart failure 2018 Med J Aust 2018; 209: 363-369.

Becker, S, Kribben A, Meister S, Jonas Diamantidis C, Unger N, Mitchell A. User profiles of a smartphone application to support drug adherence – experiences from the iNephro project. PLoS ONE 2013; 8: e78547.

Bowry ADK, Shrank WH, Lee JL, Stedman M, Choudry NK. A systematic review of adherence to cardiovascular medications in resource-limited settings. J Gen Intern Med 2011; 26: 1479-1491.

Brazier J, Ratcliffe J, Salomon J, Tsuchiya A (2016). Measuring and valuing health benefits for economic evaluation. 2nd edition, Oxford University Press, Oxford, UK.

Byambarsuren O, Sanders S, Beller E, Glasziou P. Prescribable mHealth apps identified from an overview of systematic reviews. NPJ Digital Medicine, 2018; 1: 12.

Chan AW, Tetzlaff JM, Altman DG et al., SPIRIT 2013 statement: defining standard protocol items for clinical trials. Ann Intern Med 2013; 158:200-207.

Chowdhury R, Khan H, Heydon E, Shroufi A, Fahimi S, Moore C, Stricker B, Mendis S, Hofman A, Mant J, Franco OH. Adherence to cardiovascular therapy: a meta-analysis of prevalence and clinical consequences. Eur Heart J 2013; 34: 2940-2948.

Como J. Health literacy and health status in people with chronic heart failure. Clinical Nurse Specialist Journal 2018; 32: 29-42.

Drummond M, Sculpher M, Claxton K, Stoddart G, Torrance G (2015). Methods for the Economic Evaluation of Health Care Programs. 4th edition, Oxford University Press, Oxford, UK

Forsytha P, Richardson J, Lowrie R. Patient-reported barriers to medication adherence in heart failure in Scotland. International Journal of Pharmacy Practice 2019 (In Press).

Goldstein CM, Gathright EC, Dolansky MA, Gunstad J, Sterns A, Redle JD, Josephson R, Hughes JW. Randomized controlled feasibility trial of two telemedicine medication reminder systems for older adults with heart failure. Journal of Telemedicine and Telecare 2014; 20: 293–299.

Herdman M, Gudex C, Lloyd A, Janssen M, Kind P, Parkin D, Bonsel G, Badia X. 2.Development and preliminary testing of the new five-level version of EQ-5D (EQ-5D-5L). Qual Life Res 2011; 20: 1727-1736.

Hulley SB, Cummings SR, Browner WS, Grady D, Newman TB. Designing clinical research: an epidemiologic approach. 4th ed. Philadelphia, PA: Lippincott Williams & Wilkins; 2013.

Marcum ZA, Sevick MA, Handler SM. Medication nonadherence: a diagnosable and treatable medical condition. JAMA 2013; 309: 2105-2106.

Morawski, K, Ghazinouri R, Krumme A, Lauffenburger J, Zhigang L, Durfee E, Oley L, Lee J, Mohta N, Haff N, Juusola J, Choudhry N. Association of a smartphone application with medication adherence and blood pressure control. JAMA Internal Medicine 2018; 178: 802-809.

Rand Corporation. 36-item short form survey instrument | RAND. 2015. Available at: [https://www.rand.org/health/surveys_tools/mos/mos_core_36item_survey.html. Accessed 10th April 2017](https://www.rand.org/health/surveys_tools/mos/mos_core_36item_survey.html.%20Accessed%2010th%20April%202017).

Riegel B, Lee CS, Dickson VV, Carlson B: An update on the Self-Care of Heart Failure Index. J Cardiovasc Nurs 2009, 24:485–497.

Risser J, Jacobson TA, Kripalani S. Development and psychometric evaluation of the Self-Efficacy for Appropriate Medication Use Scale (SEAMS) in low-literacy patients with chronic disease. Journal of Nursing Measurement 2007; 15: 203-219.

Sahle BW, Owen AJ, Mutowo MP, Krum H, Reid CM. Prevalence of heart failure in Australia: a systematic review. BMC Cardiovascular Disorders 2016; 16: 32.

Savarese G & Lund LH. Global public health burden of heart failure. Cardiac Failure Review 2017; 3, 7-11.

Strecher VJ and Rosenstock IM. The health belief model. In: Glanz K, Lewis FM and Rimer BK (eds) Health behaviour and health education: Theory, research and practice, 2^nd^ edn. San Francisco: Jossey-Bass, 1997, pp41-59.

Ware JE. The SF Community—SF-36® Health Survey update. Available at: <http://www.sf-36.org/tools/SF36.shtml>. (Assessed 4 April 2019)

World Health Organization. Adherence to long-term therapies–Evidence for action. 2003 Available at: <http://apps.who.int/medicinedocs/en/d/Js4883e/> (Accessed 8 February 2019)

Research team roles and responsibilities:

A. University of South Australia academics: Dr Vijay Suppiah; Dr Elizabeth Hotham

Roles and responsibilities:

1. Vijay Suppiah was over all in charge of writing the study protocol with assistance from clinicians (Christine Burdeniuk and Genevieve Gabb), pharmacists (Jessica-Chapman Goetz and Adaire Prosser) and NPS MedicineWise staff (Nerida Packham, Kitty Yu, Gamila MacRury and Chadi Tahan).

2. Supervision of research assistant - ensuring the smooth running and ethical conduct of the trial from recruitment of study participants to completion. Also to ensure that RA meets reporting deadlines with recruitment and conduct of trial with NPS MedicineWise staff.

3. Supervision of Masters student (RA) - ensuring the student meets deadlines as proposed by the Division of Health Sciences Masters program. Oversee the collection of data, analysis and preparation of thesis and manuscripts arising from the study.

4. Liaising with site PIs - reminding site PIs to refer suitable study participants to RA

5. Liaising with MedicineWise staff - Getting feedback from NPS staff with regards to Tiers 2, 3 and 3A follow ups.

B. NPS MedicineWise: Ms Nerida Packham

Roles and responsibilities:

Project Sponsor for NPS MedicineWise interventions, in collaboration with NPS MedicineWise App Team. Overall in charge of all activities done by the NPS MedicineWise team which includes data collection from app, Tiers 1, 2, 3 and 3A interventions provided by NPS MedicineWise pharmacists and liaising with the research assistant and staff at UniSA.

C. Site PIs: Dr Alicia Chan; A/Prof Margaret Arstall; Dr Christine Burdeniuk; Dr Genevieve Gabb; Ms Teena Wilson

Roles and responsibilities: Site Principal Investigator for their individual sites; Identification and referral of eligible participants; Study support

D. Associate investigators: Mr Jeffrey Briggs; Mr Tim Pearson; Mrs Terina Selkow; Mrs Adaire Prosser

Roles and responsibilities:

Identification and referral of eligible participants

E. Study RA; Masters Student: Ms Jessica Chapman-Goetz

Roles and responsibilities:

In charge of all daily activities related to the clinical trial, including liaising with the different site clinicians who will be referring patients to her for recruitment, recruitment of all study participants, familiarising participants in the interventional arm to the NPS MedicineWise application, liaising with NPS MedicineWise staff and pharmacists who will be doing intervention tiers 2, 3 and 3A, all follow ups with study participants at baseline, months 3, 6 and 9, and economic analysis. Complete the requirements of a masters program at University of South Australia.
