# Supplementary material for: Acceptability and feasibility of the NPS MedicineWise mobile phone application in supporting medication adherence in patients with chronic heart failure: Protocol for a pilot study
Source: PLoS One. 2022 Feb 4;17(2):e0263284. doi: 10.1371/journal.pone.0263284 (PMC8815969; doi:10.1371/journal.pone.0263284)
Supplement: S3 File — (DOCX) [file pone.0263284.s003.docx]

**Supporting information**

# **Table 1: Critical and non-critical medication list**

| **Class** | **Active ingredients** | **Critical vs non-critical** | **Comment** |
| --- | --- | --- | --- |
| ACE inhibitors | Captopril | Critical | Include all single AND combination products containing these active ingredients  Include both salts of perindopril (erbumine and arginine) |
|  | Enalapril +/- hydrochlorothiazide +/- lercanidipine |  |  |
|  | Fosinopril +/- hydrochlorothiazide |  |  |
|  | Lisinopril |  |  |
|  | Perindopril arginine +/- amlodipine +/- indapamide |  |  |
|  | Perindopril erbumine +/- indapamide |  |  |
|  | Quinapril +/- hydrochlorothiazide |  |  |
|  | Ramipril +/- felodipine |  |  |
|  | Trandolapril +/- verapamil |  |  |
| Angiotensin II receptor blockers | Candesartan +/- hydrochlorothiazide | Critical | Include all single AND combination products containing these active ingredients |
|  | Eprosartan +/- hydrochlorothiazide |  |  |
|  | Irbesartan +/- hydrochlorothiazide |  |  |
|  | Losartan |  |  |
|  | Olmesartan +/- amlodipine +/- hydrochlorothiazide |  |  |
|  | Telmisartan +/- amlodipine +/- hydrochlorothiazide |  |  |
|  | Valsartan +/- amlodipine +/- hydrochlorothiazide |  |  |
| Beta blockers | Bisoprolol | Critical |  |
|  | Carvedilol |  |  |
|  | Metoprolol succinate |  |  |
|  | Metoprolol tartrate |  |  |
|  | Nebivolol |  |  |
| Loop diuretics | Frusemide | Critical |  |
|  | Bumetanide |  |  |
|  | Ethacrynic acid |  |  |
| Aldosterone antagonists | Spironolactone | Critical |  |
|  | Eplerenone |  |  |
| Anti-arrhythmics | Digoxin | Critical |  |
|  | Amiodarone | Critical |  |
| Cardio-tonic agent | Ivabradine | Critical |  |
| Angiotensin receptor / neprilysin inhibitor | Sacubitril/Valsartan | Critical |  |
| Nitrates | Isosorbide mononitrate | Critical |  |
|  | Isosorbide dinitrate | Critical |  |
| Arteriolar vasodilator | Hydralazine | Critical |  |
| Anticoagulants | Warfarin | Critical |  |
|  | Apixaban |  |  |
|  | Rivaroxaban |  |  |
|  | Dabigatran |  |  |
| Sodium-glucose co-transporter 2 inhibitor | Empaglifozin | Non-critical |  |
| Calcium channel blockers | Amlodipine | Non-critical | Include all single AND combination products containing these active ingredients |
|  | Felodipine | Non-critical |  |
| Thiazide or thiazide-like diuretics | Indapamide | Non-critical | Include all single AND combination products containing these active ingredients |
|  | Hydrochlorothiazide | Non-critical |  |
| Iron | Ferrous sulphate +/- ascorbic acid +/- folic acid | Non-critical | Brand names: Ferrogradumet, Ferro Liquid, Ferrograd C, FGF, Fefol |
|  | Ferrous fumarate +/- folic acid | Non-critical | Brand names: Ferro-tab, Ferro-F |
|  | Iron polymaltose | Non-critical | Brand name: Maltofer |
|  | Iron Sucrose | Non-critical | Brand name: Venofer |
|  | Ferrous fumerate/folic acid/ascorbic acid/cyanocobalamin | Non-critical | Brand name: Ferro-sachets, Iron melts |
| Lipid lowering | Atorvastatin +/- amlodipine +/- ezetimibe | Non-critical | Include all single AND combination products containing these active ingredients |
|  | Simvastatin +/- ezetimibe |  |  |
|  | Fluvastatin |  |  |
|  | Pravastatin |  |  |
|  | Rosuvastatin +/- ezetimibe |  |  |
|  | Fenofibrate |  |  |
|  | Ezetimibe |  |  |

Protocol Version: 22/07/2019 version 1

**Funding**

This research is co-funded by the Innovation Connections Grant scheme by the Department of Industry, Innovation and Science and NPS VentureWise Pty Ltd. NPS MedicineWise was commissioned by VentureWise to deliver the project in collaboration with University of South Australia. NPS MedicineWise acknowledges funding from the Australian Government Department of Health to develop and maintain the NPS MedicineWise app.

**Author’s contributions**

As the Principal Investigators, VS, EH and NP envisaged the study and were integral to the development of the study design and protocol. The support and guidance of GG, AP and CP enabled the selection of the study setting and enhanced the 'real-world' nature of the design. JCG undertook the literature review, assisted with protocol refinement and implementation. KY provided invaluable technical support and insight to assist the delivery of mobile health through the NPS MedicineWise application. JCG, VS, EH, NP and KY were vital to the delivery of the intervention and the collection of data. Their contributions will extend to data analysis. All authors contributed to the editing of the study protocol and have endorsed the final version.

**Data Management**

**University of South Australia:**

Participants’ names, addresses and smart phone numbers will be collected for the purpose of intervention delivery and participant interviews. All data collected from participants will otherwise be stored electronically as de-identified data which will be stored separately from identifiable data. UniSA offers a Research Data Storage solution which enables researchers to manage, collaborate and share data with others. The UniSA Research Data Storage solution provides high-capacity storage, with data protected through daily backups and via data replication technologies. All UniSA staff members of the research team can log in and access the data using their UniSA log ins. External collaborators can be granted access with secure login through the web interface.

**NPS MedicineWise:**

NPS MW takes privacy and confidentiality seriously. In dealing with personal information, NPS MW complies with the Privacy Act and any relevant State and Territory privacy legislation. NPS MW has an Information Security Management Policy and a Risk Management Framework in place. NPS MW is Information Security Registered Assessors Program (IRAP) certified. IRAP is a multi-staged certification process that starts with a security assessment, involves a system security plan, a security risk management plan, and an incident response plan. IRAP certification ensures best practice in information security. NPS MW also has a Risk Management Committee and specific procedures in place regarding managing privacy and security. NPS MW is an ISO 9001:2018 certified organisation and has strict processes in place regarding privacy and security of data. Certification is the provision by an independent body of written assurance (a certificate) that management system meets specific requirements. Certification is also known as third party conformity assessment. This is a way of showing outsiders that the organisation has an effective quality management system in place.

Since July 2010, NPS MedicineWise has delivered a national in-house Medicines Line telephone service for Australian consumers. As enquiries with Medicine Information Pharmacists on Medicines Line are considered a medical record, details of each call are recorded in the secure software system MiDatabank^®^. MiDatabank^®^ is a Windows software application that enables Medicines Information staff (often pharmacists) in an organisation to record, manage, store, and search their enquiries. The MiDatabank^®^ database is only accessible via software on NPS MedicineWise in house computers and not available to team members offsite from the organisation. MiDatabank^®^ is protected with personalised login and passwords. During the Medicine Adherence in Chronic heart Failure trial Tier 3 intervention participant data will be stored in MiDatabank^®^.

**Monitoring**

A data monitoring committee was not deemed necessary as the study is of relatively short duration (6 months) and aims to evoke behaviour change through a low-risk, mHealth-based, tiered pharmacist intervention.

Due to the real-world nature of the intervention, data analysis will occur upon trial completion. The trial will be terminated once the 6-month follow-up has been conducted for the last recruited participant.

**Harms**

Solicited or spontaneously reported adverse events and other unintended effects of the trial intervention will be recorded and immediately reported to the participant’s treating physician (site Principal Investigator) for review and management as per standard of care. Given the nature of the intervention, participants are expected to be a low risk of harm due to taking part in the study.

**Protocol amendments**

If significant modifications to the protocol are required that may affect the benefit vs harm profile for actual or potential participants, or the study aims, design, intervention, and processes/procedures, then amendments will be submitted to the Central Adelaide Clinical and University of South Australia Human Research Ethics Committees for approval. Any alteration/s to the existing protocol will occur in consultation with researchers from NPS MedicineWise and will be communicated in a timely manner to site Principal Investigators (cardiologists), cardiology nurses and cardiology pharmacists. Where relevant, trial participants will be informed of protocol modifications and re-consented, as necessary.
